# Effects of cadmium chloride on the cultured human lens epithelial cells

**Published:** 2012-04-19

**Authors:** Nang-Hee Song, Jae-Woong Koh

**Affiliations:** Department of Ophthalomology, Chosun University College of Medicine, Gwangju, Republic of Korea

## Abstract

**Purpose:**

To investigate cadmium chloride cytotoxicity in human lens epithelial cells as well as the mode of cell death and its mechanism.

**Methods:**

Cultured human lens epithelial cells were challenged with cadmium chloride. Morphological changes of human lens epithelial cells caused by cadmium chloride exposure were evaluated by microscope. Cell viability was evaluated by the 3-(4,5-dimethylthiazol-2-yl)-2,5-dipheny tetrazolium bromice (MTT) assay. To explore the mechanism of cell death, p53 and caspase-8 levels were measured by western blotting.

**Results:**

Microscopic examination indicated that cell death increased after cadmium chloride exposure compared to untreated cells. The MTT assay demonstrated that cadmium chloride significantly decreased cell viability in a dose dependent way. Western blot and quantitative analysis showed that both p53 and caspase-8 increased after cell exposure to cadmium chloride. p53 increased 210% and caspase-8 increased 30% in the experimental group as compared with the control group.

**Conclusions:**

Cadmium chloride induced cytotoxicity and apoptosis in human lens epithelial cells and the mechanism of apoptosis involve an increased expression of p53 and caspase-8.

## Introduction

Cadmium is one of the most notorious heavy metals and one of the members of the USA Environmental Protection Agency’s “Priority List of Chemicals,” has been classified by the International Agency for Research on Cancer as a human carcinogen [1]. Tobacco smoke is the highest source of exposure in the general population due to absorption of cadmium by the lungs [2,3]. Therefore, human exposure to cadmium is essentially unavoidable. Because of the its biologically long half–life which has been estimated to be 10–30 years in humans, cadmium has been demonstrated to cause pathological changes in organs such as liver, brain, kidney, and lung [4]. It also accumulates in various ocular tissues such as the lens, retina, ciliary body, and vitreous humor [5]. Large amounts of cadmium have been detected in lenses of chronic smokers who also exhibit early cataract formation [6]. And increased cadmium levels have also been reported in cataracts compared to clear human lenses [7-9].

The studies clearly show that there is accumulation of the heavy metal ion of cadmium in the lens of chronic smokers which might have a role in cataractogenesis [7-9]. According to Ramakrishnan et al. [9] 40–80 μM levels of cadmium, which are approximately the levels found to be present in cataracts of smokers and cadmium may hasten cataractogenesis directly by interaction with lens proteins and indirectly by its competition with copper, zinc, and selenium and causing a decrease of antioxidants. Recent data show a fourfold increase in heavy smokers (15.4±0.4 mol/g) and a nearly threefold increase in light smokers (10.1±0.4 mol/g) as compared to non-smokers (3.7±0.9 mol/g) [10].

However, the mechanism of cigarette smoke-induced lenticular opacities is poorly understood. The normal single layer-cuboid shaped lens epithelial cells are essential for maintaining the metabolic homeostasis and transparency of the entire lens [11]. If lens epithelial cell viability is required for transparency, then with lens epithelial cell death, the lens will become opaque [12]. They contain the highest levels of enzymes and transport systems in the lens and are the first part of the lens exposed to environmental insults [13]. Under normal physiologic conditions, most of these cells have a relatively long life span [14]. But if such conditions are altered or disturbed these maintenance functions may be jeopardized, possibly resulting in opacification of the lens [14].

Recently, both in vitro and in vivo studies have shown that treatment of adult lens with stress factors induces apoptosis of lens epithelial cells, which is followed by cataractogenesis [15-17]. Damaged lens epithelium will be leaky to calcium. The influx of calcium into the underlying fiber cells can activate the cellular cysteine protease calpains and caspase [18,19], which then degrade cytoskeleton components [19,20] and lens crystallins [21,22]. These processes eventually lead to crystallin aggregation [23], which together with other changes such as uptake of water and electrolytes lead to development of cortical and nuclear cataract [24,25]. But data on the mechanism of apoptosis in human lens epithelium from cataractous lenses are scarce and conflicting.

We hypothesized that cadmium, a major smoke constituent, could cause cataractous changes in the lens through lens epithelial cell damage and explored the mechanism of apoptosis that occurs in a cultured human lens epithelial cell line after exposure to cadmium. We also investigated whether cadmium-induced apoptosis was related to activation of p53 and caspases-8. p53 can induce apoptosis, cell cycle control and DNA repair in response to cellular stress.

## Methods

### Culture of cells

The human lens epithelial line CRL 11241 (B-3 cell, ATCC, Rockville, MD) was used for this study. These cells were cultured in Modified Eagle's Medium (MEM; Sigma, St Louis, MO), supplemented with 10% fetal bovine serum (FBS), 100 U/ml penicillin, 100 μ/ml streptomycin, and 25 μg/ml nystatin, and were cultured at 37 °C in humidified atmosphere, containing 5% CO_2_. This research adhered to the tenets of the Declaration of Helsinki and was approved by the institutional review board (IRB) of the Chosun Medical School.

### Morphological observation of human lens epithelial cells

Cultured human lens epithelial cells were placed in 6 well plate (5×10^4^ cells/ml), after 24 h incubation, exposed to 80 μM of cadmium chloride (CdCl_2,_ Catalog No. 202908; Sigma) for 4 h. The control group and experimental group were then observed using a contrast-phase microscope (Leica, Wetzlar, Portugal).

### Measuring the effect of cadmium on cell viability in human lens epithelial cells

Cytotoxicity was determined by an MTT (3-(4,5-dimethylthiazol-2-yl)-2,5-diphenyl-tetrazolium bromide; Sigma) assay. The human lens epithelial-B-3 cells were placed in a 96 well plate (2×10^4^ cell/ml) overnight and then exposed to various concentrations of cadmium chloride (0–100 μM) dissolved in PBS. Cells incubated without cadmium served as control group. Twenty four hours after incubation with cadmium, cell viability was evaluated using the MTT assay. In this assay, MTT is reduced to purple formazan in the mitochondria of living cells. A solubilization solution is added to dissolve the insoluble purple formazan product into a colored solution. The MTT assay was performed using a standard protocol and optical density was measured at 570 nm using a spectrophotometer.

### Western blot

Both control groups and experimental groups (exposed to 60 μM/ml of cadmium chloride) were evaluated by western blot analysis. Briefly, after cadmium treatment human lens epithelial cells were washed with Dulbecco’s PBS 1 time and cells were collected. SDS-loading buffer (100 μl; 50mM Tris-HCl ; pH6.8, 2% SDS, 0.1% Bromphenol blue, 10% glycerol) was added to the collected cells and electrophoresis was conducted. Electrophoresis was done at 100 V in Tris buffer solution (pH8.8, 0.025 M Tris, 0.192 M glycine, 0.1% SDS) using a 30% polyacrylamide gel. After protein separation by electrophoresis the proteins were transferred to nitrocellulose membrane. The membrane was then blocked in 5% non-fat milk in Tris buffered saline (TBS: 0.1% Tween-20 in pH7.4 Tris-based saline buffer) for 1 h at room temperature and washed with TBS twice. The membrane was incubated with primary antibody diluted to 3/1000 in 5% non-fat-milk-TBS. Anti-rabbit polyclonal Anti-p53 Ab, anti-caspase-8 Ab were used as the primary antibodies. After washing with TBS 4 times, incubation with secondary antibody combined in horseradish peroxidase (goat anti-mouse IgG) diluted to 3/1,000 in 5% non-fat-milk-TBS was performed for 1 h. Immunoreactive bands were visualized using an enhanced chemiluminescence light detection kit (Amersham, Piscataway, NJ). Beta-actin (GeneTex Inc., San Antonio, TX) was used as an internal control. In all of the figures with densitometry data, optical density refers to the integrated density.

### Analysis of experimental results

To increase the reliability of the data, all experiments were repeated 3 times and average values were calculated. SPSS ver. 10.1 (SPSS Inc., Chicago, IL) was used to compute routine statistics. The data were analyzed for significance using repeated measures by two-way ANOVA, followed by a Duncan’s multiple range test of post hoc tests, and were expressed as a mean percentage of the control value plus SEM. A p value <0.05 was considered significant.

## Results

### Morphological changes of human lens epithelial cells by cadmium chloride

**Figure 1:**
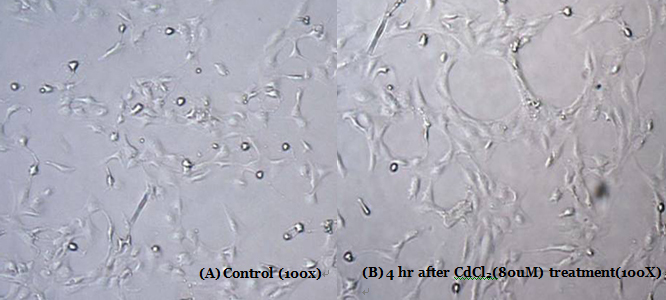
Morphological changes of human lens epithelial cells after exposure to cadmium chloride. Photograph of the human lens epithelial cell control group (**A**) and the experimental group (human lens epithelial cells exposed to 80 μM of cadmium chloride; **B**). Compared to untreated cells, microscopy analysis indicated that cell damage increased after cadmium chloride exposure.

Microscopic observation of the human lens epithelial cells exposed to 80 μM cadmium chloride revealed marked morphological changes. It looked damaged in cadmium chloride treated cells (Figure 1).

### Effect of cadmium concentration on cell viability

**Figure 2:**
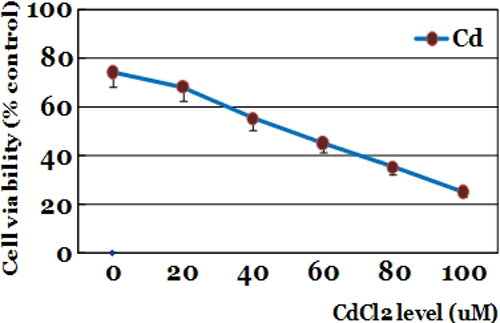
Effect of cadmium on the cell viability of human lens epithelial cells. The human lens epithelial cells cells were exposed to various concentration of cadmium chloride. Twenty four hours after incubation, cell viability was evaluated by the MTT assay. Optical density was measured at 570 nm using a spectrophotometer. Cadmium chloride significantly decreased cell viability in a dose dependent way.

Cadmium chloride significantly decreased cell viability in a dose dependent way (Figure 2). Cell viability was 74.5±1.6% in control group, 68.7±4.1% in 20 μM cadmium chloride, 55.9±3.3% in 40 μM cadmium chloride, 45.1±3.3% in 60 μM cadmium chloride, 35.4±3.6% in 80 μM cadmium chloride, and 25.6±2.4% in 100 μM cadmium chloride. Cell viability decreased to less than 50% in 60 μM cadmium chloride (p<0.05).

### Western blot analysis

**Figure 3:**
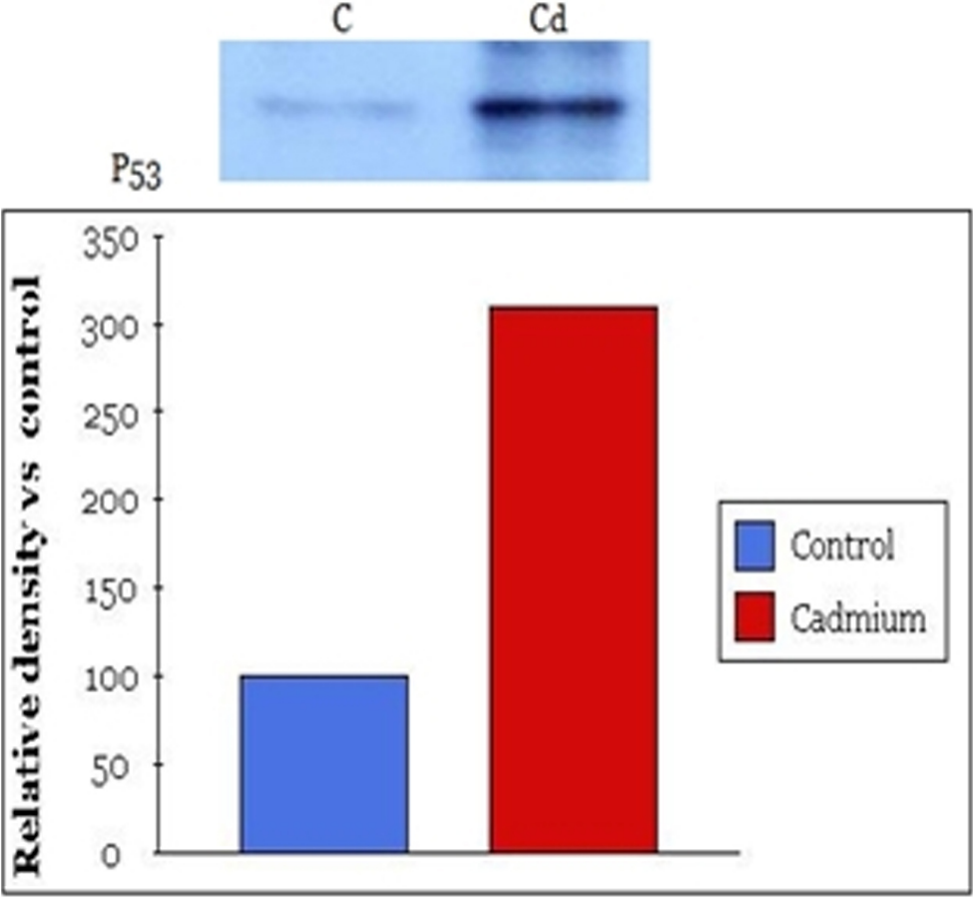
Effects of cadmium chloride on the expression of p53 in human lens epithelial cells. Western blot and quantitative analysis showed that p53 increased after exposure to 60 μM of cadmium chloride. p53 increased 210% in the experimental group as compared to the control group.

**Figure 4:**
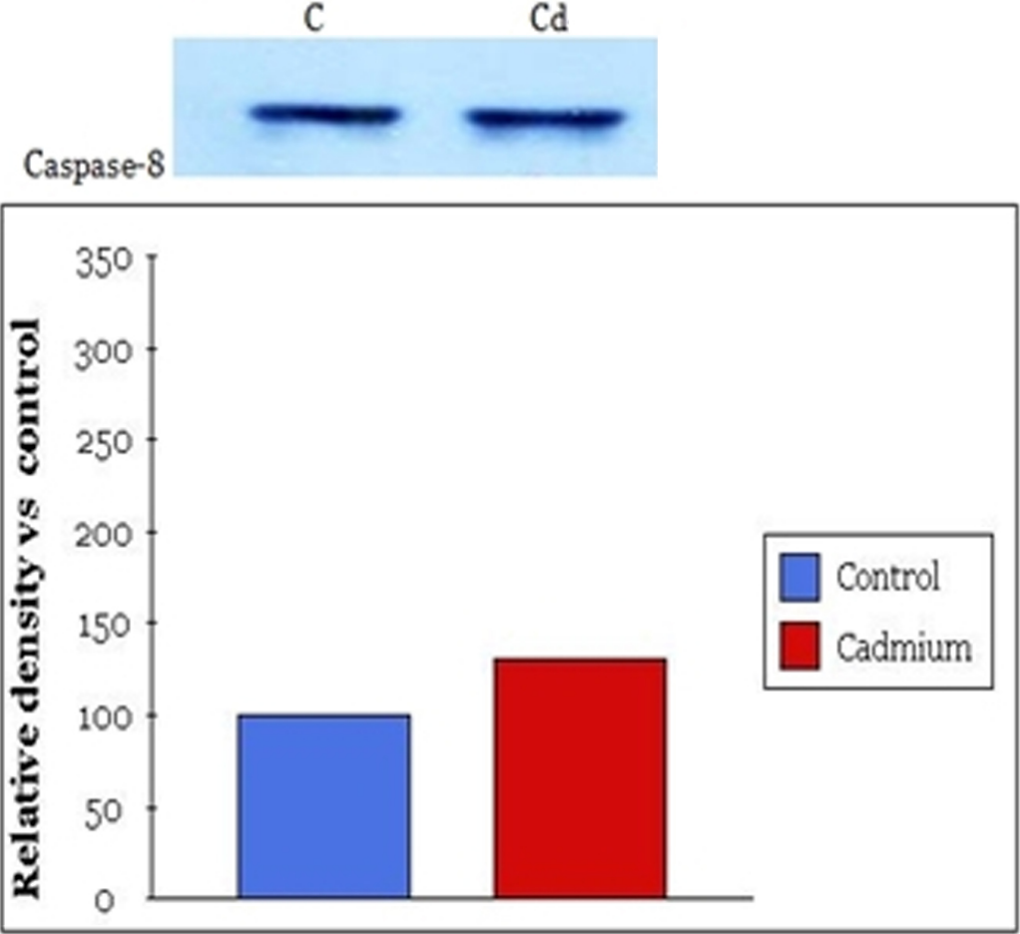
Effects of cadmium chloride on the expression of caspase-8 in human lens epithelial cells. A representative western blot and quantitative analysis showed that caspase-8 increased after exposure to 60 μM of cadmium chloride. Caspase-8 increased 30% in the experimental group as compared to the control group.

Western blot and quantitative analysis showed that p53 increased in lens epithelial cells after exposure to 60 μM of cadmium chloride. p53 increased 210% in the experimental group compared to the control group (Figure 3). Western blot and quantitative analysis showed that caspase-8 also increased after exposure to 600 μM of cadmium chloride. Caspase-8 increased 30% in the experimental group compared to the control group (Figure 4). A single band of 53 kDa and 55 kDa corresponding to p53 protein and caspase 8, respectively, were present in lens epithelial cell lysates.

## Discussion

Cigarette smoke contains 4,000 identified chemical compounds and of these toxic materials are heavy metals, particularly cadmium, which go into our system through inhalation of smoking [26]. It readily pass into the bloodstream and may accumulate in specific organs [26]. Indeed smoking has long been considered a major source of several heavy metals in blood and various organs and cadmium is regarded as one of the “strong carcinogens” in tobacco smoke [27]. Cadmium has been found in several studies consistently to transfer into the smoke phase, which coupled with the fact that the tobacco plant is particularly efficient in accumulating cadmium from the soil and translocating most of the metal to the leaves makes this element the prime focus for particular investigation for any potential toxic effects [28,29].

Cadmium exemplifies the double edge nature of many toxic substances [30]. On the one hand, it can act as a mitogen, stimulate cell proliferation, inhibit apoptosis, inhibit DNA repair, and promote cancer in several tissues. On the other hand, it causes tissue damage by inducing cell death [31]. According to Templeton and Liu [31], the concentration dependence of the effects of cadmium is an important factor for cell death. There was also a report that low dose exposure is related to various types of apoptotic cell death and high dose exposure is related to necrosis [31]. That is, depending on the exposure conditions, cadmium may induce either necrosis or apoptosis in mammalian cells.

Cadmium is a direct enzyme poison. Cadmium inhibits plasma membrane calcium channels and Ca^2+^ ATPase groups and hence can inhibit enzymes. It can exerts toxic effect [32]. Cadmium is also a potent oxidative stress factor [33]. Oxidative stress occurs when the levels of pro-oxidants exceed the ability of the cell to respond through antioxidant defense [33]. Oxidative stress is a crucial event in activation of apoptotic mechanisms [33]. Cadmium induces excessive reactive oxygen species (ROS) generation and it may alter the structure and function of proteins, lipids and DNA, besides activating various signaling pathways which collectively cause apoptosis [34,35]. One of the major effects that oxidative stress induces is the death of lens epithelial cells [36,37]. According to Kalariya et al. [38] Cadmium can increase ROS levels in human lens epithelial cells and weakens antioxidative reactions by inhibiting the action of peroxide removal enzymes. Thus, both oxidative damage and direct toxicity induced by cadmium appear to play major roles in cataract formation. Recently, cadmium was found to induce DNA fragmentation, a biochemical hallmark of apoptosis, in cultured renal cells, hepatocytes and human T-cells [39].

Therefore, the various toxicities of cadmium are thought to be caused by the induction of apoptosis. Cadmium modulates protein kinase, phosphatase activities and tranascription factors and mitogen-activated protein kinase (MAPK) [40,41]. Mitochondria, caspases and ROS pathways all seem to palys role in cadmium induced apoptosis [42]. It is conceivable that cadmium may induce different apoptotic pathways in different cell types depending on the exposure conditions. But the apoptotic pathway induced by cadmium remains controversial. A large number of studies have demonstrated that cadium-induced activation of the MAPK pathway leads to apoptosis in various cell types [34,35,38]. According karariya et al., the activation of MAPK pathway along with ROS generation and apoptosis in human lens epithelial cells could collectively damage the lens epithelial layer which could make the lens vulunerable to develop opacity [38]. Toxic metals have been reported to induce the generation of reactive oxygen species, which may target the mitochondrial membrane, triggering one or more of the intrinsic, mitochondrial apoptotic pathways leading to activation of pro-caspases-9 and-3 [43]. However, ROS are also thought to play a role in the Fas receptor-mediated, extrinsic apoptotic pathway via c-jun N- terminal kinase (JNK) mediated induction of FasL or Fas expression [44]. Recently, it was demonstrated that toxic metal- induced apoptosis in cultured murine podocytes through the extrinsic Fas-associated death domain protein (FADD) capsase 8 pathway, rather than the mitochondrial apoptotic pathway [44].

Apoptosis is an important mechanism to maintain homeostasis in multicellular organisms and is a series of controlled processes that selectively removes damaged cells without injuring surrounding tissues [45]. Apoptosis is a normal morphogenetic process of lens development [46]. During development, apoptosis is necessary for lens vesicle formation and detachment [46]. And apoptosis helps to remove damaged epithelial cells or aberrantly differentiated lens cells [46]. Suppression or enhancement of developmental apoptosis because of genetic mutations and manipulations, or environmental conditions causes formation of abnormal lenses or absence of the ocular lens [46]. It is a very sophisticated operative process and the mechanism can be largely divided into an external mechanism and internal mechanism [47]. The signal in the external mechanism (receptor–dependent pathway) starts from death receptors, such as Fas or tumor necrosis factor-α (TNF-α) receptors, and is passed to caspase-8 which activates caspase-3, the caspase that directly triggers apoptosis [47]. This external signal can cause apoptosis through the internal mechanism by increasing transcription of B-cell lymphoma protein-2 (Bcl-2) family proteins and stimulating mitochondria at the same time [47]. The internal mechanism (mitochondria–dependent pathway) activates caspase-9 through apoptotic protease activating factor-1 (Apaf-1) by cytochrome C being secreted in mitochondria and this causes death of cells by activating caspase-3 and Bcl-2 family protein regulates apoptosis by causing secretion of cytochrome C by adjusting the permeability of mitochondria [48]. Caspases can be broadly dived into two groups: initiator caspases, such as caspases-8,-9 and −12, whose main function is to activate downstream caspases, and executor caspases, such as caspases-3,-6 and −7, which are a responsible for degradation of cellular proteins [49]. Caspase-8, encoded by the *CASP8* gene, is an initiator caspase of the death receptor pathway, as well as a target of the caspase-3 downstream pathway of mitochondria, and is composed of 60 amino acids of NH_2_-terminal death effector domain (DED), that facilitates caspase-8-FADD direct interaction. Depending on the cellular context, this results in different outcomes [49]. Cell death induced through the p53 pathway is executed by the caspase proteinases [49].

In this study, the toxicity of cadmium in human lens epithelial cells was measured by MTT method after culturing the cells in medium containing 20 μM 40 μM, 60 μM, 80 μM, and 100 μM of cadmium chloride. It was confirmed that cytotoxicity increased significantly with increasing concentrations. To explore the mechanism of the apoptotoic process, the expression of p53 and caspase-8, a potent mediator of apoptosis, were examined. Western blot analysis revealed that protein expression levels of p53 and caspase 8 increased by 210% and 30%, respectively, in the group exposed to cadmium compared to the control group. Thus, apoptosis in the human lens epithelial cells is related to p53 and caspase-8 expression. Based on the result above, cadmium affects cytotoxicity and death of human lens epithelial cells via a p53 dependent pathway and activation of caspase-8. How cadmium activates caspase-8 is not clear, but it’s clear that cadmium has an effect on caspase-8 protein levels, suggesting that the death receptor pathway might contribute appreciably to the observed cadmium induced apoptosis. Since it is difficult to obtain normal human materials, cultured models have been used to further examine the relationship between apoptosis and cataractogenesis. In this study, we confirmed that cadmium induced apoptosis occurs in cultured human lens epithelial cells, but great caution should be useded in transferring this finding to the human sitiuation. because there are a few difference in lens composition between them, cultured human lenses have a very low level of crystallins.

In conclusion, we studied the effects of cadmium on the viability of human lens epithelial cells and showed that cadmium caused significant decline in the viability of human lens epithelial cells in a dose dependent manner. Cadmium also induced p53 and caspase-dependent apoptosis of human lens epithelial cells, a potential cause of human lens opacity.
